# A Practical Treatise on the Diseases of Children

**Published:** 1886-02

**Authors:** 


**A Practical Treatise on the Diseases of Children.** By ALFRED VOGEL, M. D., Prof. of Clinical Medicine in the University of Dorpat, Russia. Translated and edited by H. Raphael, M. D., formerly House Surgeon to Bellevue Hospital. Third American, from the eighth German edition. Revised and enlarged, illustrated with lithographic plates. New York: D. Appleton & Co., 1, 3 and 5 Bond street. 1885.

Vogel's Treatise on Diseases of Children has a world-wide reputation, having appeared in the Russian, German, Dutch and English languages. This is a deserved success, for it is a book admirably adapted to the wants both of the practitioner and student. The present edition is brought well up to the present state of pathological knowledge, it is complete without prolixity, and the book bears upon its pages the evidence of the work of a skillful and experienced clinical practitioner. The diagnoses and differential relations of diseases to each other are accurately described, and the therapeutics judicious and discriminating. We would most heartily commend the book as one of the most valuable upon the subject, and, indeed, few physicians can afford to forego the advantages to be derived from the possession of this work.

